# Smart wearable biosensors: a transformative synergy between diagnosis and treatment of disease

**DOI:** 10.1039/d6ra02749k

**Published:** 2026-07-24

**Authors:** Yachana Misha, Radheshyam Jena, Aman Shukla, Darshan R. Telange, Alaa A. A. Aljabali, Vijay Mishra

**Affiliations:** a School of Bioengineering and Biosciences, Lovely Professional University Phagwara (Punjab)-144411 India; b School of Pharmaceutical Sciences, Lovely Professional University Phagwara (Punjab) 144411 India vijaymishra2@gmail.com; c Datta Meghe College of Pharmacy, Datta Meghe Institute of Higher Education and Research (DMIHER) (DU) Sawangi Meghe Wardha (Maharashtra) 442001 India; d Department of Pharmaceutics & Pharmaceutical Technology, Yarmouk University Irbid Jordan alaaj@yu.edu.jo

## Abstract

Smart wearable biosensors represent a significant paradigm shift from one-time sample analysis to real-time biochemical monitoring at the body interface. Besides the flexible design of the device or wireless readout, their clinical utility will also require the reliability of the entire sensing pathway under real physiological conditions. This pathway involves biofluid access to clinical interpretation. Despite rapid progress, many wearable biosensor platforms remain limited by weak biofluid–blood correlation, receptor degradation, biofouling, motion artefacts, sensor drift and insufficient patient-level validation. Thus, a chemistry-to-clinics approach is crucial to assess the analytical reliability and translational readiness of recognition elements, sensing materials, and engineered biointerfaces. Enzymes, antibodies, aptamers, nucleic-acid systems, molecularly imprinted polymers, and nanozymes are discussed within the context of selectivity, stability, antifouling behaviour and suitability for continuous monitoring of sweat, interstitial fluid, tears, wound exudate and breath condensate. The functionality of carbon nanostructures, metal-based nanomaterials, hydrogels, MXenes, metal–organic frameworks and self-powered interfaces are evaluated in terms of their applications in amplification, mechanical conformity, biofluid handling and signal stability. Artificial intelligence is positioned as a support layer for signal correction, calibration, classification, multimodal fusion and predictive interpretation, rather than as a substitute for robust sensing chemistry. This review provides a critical chemistry-to-clinical perspective on smart wearable biosensors and outlines the validation, manufacturing, cybersecurity, post-market surveillance and benchmarking requirements needed for their translation into reliable diagnostic and therapeutic-monitoring technologies.

## Abstract 2

Smart wearable biosensors represent a significant paradigm shift from one-time sample analysis to real-time biochemical monitoring at the body interface.

## Graphical abstract

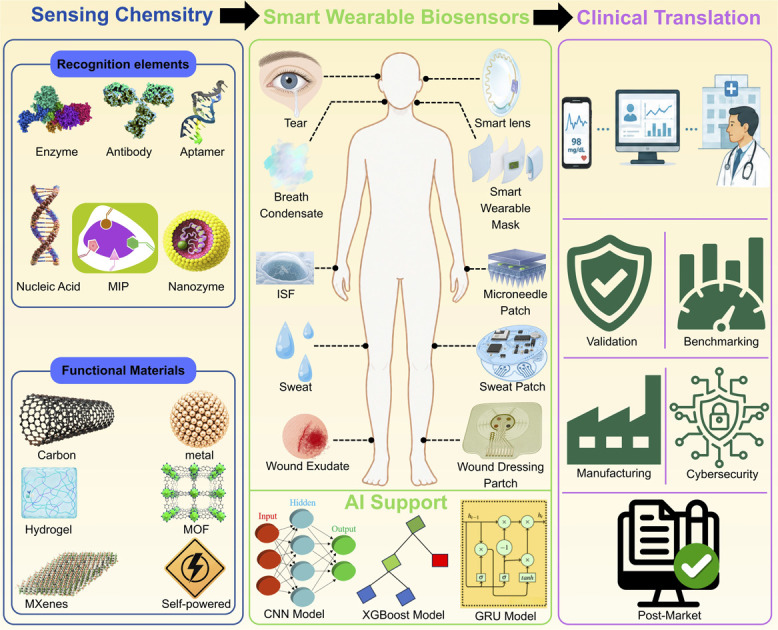

## 1 Introduction

Smart wearable biosensors are emerging as next-generation analytical platforms that extend conventional biosensing from isolated sample testing to continuous, body-integrated biochemical monitoring.^1^ Conventional biosensors have been crucial for the laboratory diagnosis and point-of-care testing of clinically relevant analytes such as glucose, lactate, proteins, nucleic acids, pathogens, hormones, inflammatory mediators, and therapeutic drugs.^2^ However, their reliance on collected samples, single-point measurements, trained handling and delayed feedback restricts their ability to detect real-time fluctuations in markers of disease development, physiological stress and therapeutic response.^3^ Smart wearable biosensors overcome this limitation with selective biochemical recognition, flexible materials, engineered biointerfaces, miniaturized transducers, wireless communication, and intelligent data interpretation, all in skin-compatible, minimally invasive or implantable formats.^4^ Their novelty is not only in their wearability, but also in their potential to enable a dynamic, patient-centred, and longitudinal monitoring strategy.^5^

The present work is focused on smart wearable biosensors as high-end platforms beyond the conventional biosensing systems, stressing biochemical recognition, materials chemistry, sensing interfaces, transduction mechanism, disease applications, AI-assisted signal interpretation, and clinical translation.^6^ Biochemical and chemical recognition elements, including enzymes, antibodies, aptamers, nucleic acid probes, CRISPR-based systems, molecularly imprinted polymers, cells, microbes, and nanozymes, are discussed in relation to their selectivity, stability, reusability, and suitability for wearable environments.^7–9^ These recognition systems allow metabolites, proteins, hormones, nucleic acids, drugs, inflammatory markers, molecules of infection, and cancer associated biomarkers to be detected from readily accessible biofluids like sweat, tears, saliva, interstitial fluid, wound exudate, and breath condensate.^10^ Compared with conventional sample-based testing, the use of these biofluids allows more frequent, non-invasive, or minimally invasive monitoring of biochemical changes under real-life conditions.

The performance of smart wearable biosensors hinges on materials chemistry and biointerface engineering. Carbon-based materials like graphene, carbon nanotubes, and carbon nanofibers increase electron transfer, surface area and conductivity.^11^ Catalytic activity, flexibility, porosity, bioreceptor immobilization and signal amplification are enhanced by metallic nanoparticles, metal oxides, conductive polymers, hydrogels, MXenes, metal–organic frameworks, covalent organic frameworks and other 2D materials.^12,13^ Conformal contact with the skin or other biological surfaces is possible with flexible substrates such as paper, textiles, elastomers, or tattoo-like films.^11^ Additionally, during long-term use, interfaces for antifouling, adhesive, and biocompatibility must be provided to minimize signal drift, biofouling, irritation, mechanical instability, and loss of sensor accuracy.^14^ Thus, the clinical reliability of smart wearable biosensors is achieved *via* the synergy in the design of the recognition chemistry, sensing material, biointerface, and transduction mechanism.^15^

Smart wearable biosensors are widely used in diagnosis and monitoring of disease for therapeutic purposes. Sweat-based platforms can be used to monitor the metabolic state, stress, electrolyte, and hydration status, whereas microneedle biosensors can be used to create minimally invasive access to the interstitial fluid for glucose, drug, hormone and biomarker measurement.^16,17^ Smart contact lenses allow for monitoring the eye and body from the tear fluid, while textile, electronic tattoo and skin-patch biosensors allow for continuous biochemical surveillance.^2,18^ In addition, implantable, multiplexed and lab-on-a-chip wearable biosensors have been extended to cancer, cardiovascular disease, infectious disease, wound healing, inflammation, sepsis risk, post-treatment monitoring, therapeutic drug monitoring, closed-loop therapy, reproductive health, transplant monitoring, chemotherapy or radiotherapy toxicity evaluation, and image-readable biosensing platforms.^19–21^

The use of Artificial Intelligence (AI) to improve signal processing functions without the necessity for replacing the sensing chemistry of smart wearable biosensors can be very beneficial. AI-driven techniques can diminish noise, fix motion artifacts, address sensor drift, detect irregular biomarker patterns, combine multimodal biosensor information, and trigger predictive alerts.^8,22^ However, interpretation with AI still relies on high-quality data, clinically meaningful biomarkers and stable materials, in addition to validated sensing mechanisms. There are still significant challenges with respect to translation, such as analytical validation, reproducibility, long-term stability, calibration, user comfort, privacy, regulatory approval, manufacturing scale-up and standardised benchmarking.^23,24^ Overall, smart wearable biosensors represent a chemistry-driven and clinically relevant evolution of conventional biosensors, offering new opportunities for continuous disease diagnosis, therapeutic monitoring, and personalised healthcare.

## 2 Current technological advancements and trends in smart wearable biosensors

A biosensor is an analytical device composed of a biorecognition element and a transducer, where the bioreceptor selectively interacts with a target analyte and the transducer converts this biochemical event into a measurable physicochemical signal.^25^ The progress in biosensor development has shifted from traditional diagnostic platforms to small, flexible, and compatible body platforms for practical health monitoring.^26^ This shift has been enabled by progress in electrochemical sensing, microfluidic handling, nanostructured materials, flexible substrates, and wireless readout technologies.^27,28^ The present trend indicates that biosensors are no longer limited to discrete sampling but are now being developed for continuous biochemical monitoring in the form of wearable and implantable devices.^29^ Smart wearable biosensors further extend this concept by continuously monitoring biochemical and biophysical signals in accessible biofluids and by using multimodal sensing and AI-assisted signal processing to improve interpretation.^1^ These developments are crucial as they enable the integration of analytical performance with user-centred monitoring, remote healthcare and personalized disease management.^30^

### 2.1 Evolution and emerging trends in smart wearable biosensors

**Fig. 1:**
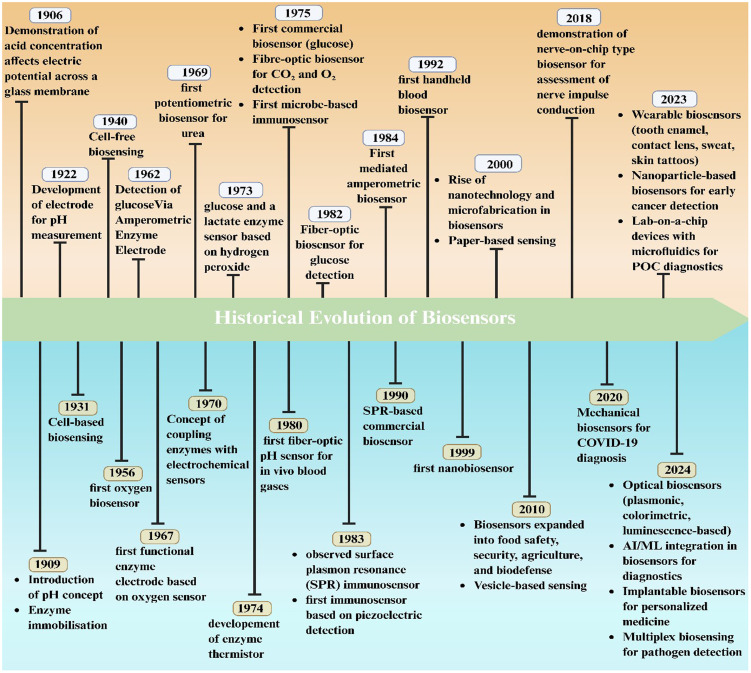
Timeline of advancement in biosensors.

The development of biosensors initiated with the classical biorecognition-transducer systems, in which enzymes, antibodies, nucleic acids, or receptor-based elements detect biological interactions and transform them into measurable output. These early platforms established the basis of clinical and point-of-care diagnostics, but most of them depended on collected samples and instrument-assisted analysis. Subsequent advances in nanomaterials, microfluidics, electrochemical interface, optical sensing and flexible fabrication techniques advanced the level of sensitivity, lowered sample quantities, and enabled device miniaturization.^27,31^ These developments laid the technological foundation for connecting the traditional biosensors with the wearable systems. The historical milestones are shown in the timeline represented in Fig. 1.

The field has recently moved towards smart wearable biosensors working directly on or in the body. Wearable sweat sensors are now available for the continuous monitoring of metabolites, nutrients, electrolytes and other biochemical markers at rest and during exercise.^26^ The textile-integrated biosensors, electronic tattoo-like sensors, and flexible skin patches continue the evolution of biosensors into soft, conformable, and user-friendly formats.^32^ Another emerging approach for non-invasive monitoring of the eye and systemic conditions is smart contact lenses and tear-based biosensors.^33^ Microneedle and insertable biosensors further expand the field by accessing interstitial fluid or implanted sensing sites for minimally invasive long-term monitoring.^29,34^

The most significant trend is the shift from detection to integrated biochemical monitoring. Biosensors, that are wearable or implantable, are now being integrated with microfluidic sampling, wireless communication, soft materials, and miniaturized electronics for real-time data collection outside of traditional clinical environments.^28,30^ Flexible, stretchable, and biocompatible biosensors with enhanced structural design and device integration are being pursued using additive manufacturing and 3D printing technologies.^35,36^ Therefore, the advancement of biosensors cannot be considered solely in terms of the miniaturization of devices, but as a shift from sample analysis to body-fitted devices that can monitor biochemical changes over time.

### 2.2 Comparative perspective on conventional and smart wearable biosensors

Conventional biosensors have made a huge contribution to disease diagnosis by providing the ability to detect the biomarker in an environment of controlled assay conditions, such as glucose analysis using mediator-based enzyme electrodes.^37^Table 1 illustrates the major differences between conventional biosensors and smart wearable biosensors. However, such systems generally provide episodic diagnostic information, whereas smart wearable biosensors integrate flexible, skin-interfaced or sweat-based platforms for real-time monitoring of metabolites, stress biomarkers, and physiological variations.^38–40^ This transition highlights the novelty of wearable biosensors as continuous, personalized, and body-integrated diagnostic systems rather than isolated testing platforms.

**Table 1:** Conventional biosensors *versus* smart wearable biosensors

Parameter	Conventional biosensor	Smart wearable biosensor	Improvement offered by smart wearable biosensors	References
Diagnostic timing	Using a paper-based vertical flow assay in a single patient sample, levels of serum cardiac injury markers such as myoglobin, CK-MB and heart-type fatty acid-binding protein can be quantified	A microneedle-integrated capacitive biosensor enables minimally invasive, *in situ* monitoring of cardiac troponin I from interstitial fluid using antibody-functionalized microneedles	Migrate cardiac injury assessment from centralized blood testing to fast, minimally invasive biomarker monitoring at the body interface	41 and 42
Biofluid used for diagnosis	Inflammatory markers in serum or whole-blood are typically measured using electrochemical CRP immunosensors	A wearable, wireless patch can measure C-reactive protein in sweat	Lowers reliance on invasive blood sampling and enables repetitive inflammatory monitoring in everyday conditions	43 and 44
Testing environment	SARS-CoV-2 lateral flow immunoassays require collected respiratory or clinical samples to be applied to a test strip	A face-mask-integrated CRISPR biosensor can detect SARS-CoV-2 nucleic acid signatures from breath-associated exposure within the mask	Moves infectious-disease biosensing closer to the patient's natural breathing environment and reduces the need for separate sample handling	45 and 46
Monitoring frequency	Blood lactate biosensors measure lactate after blood sampling and provide a result at the time of testing	A temporary tattoo lactate biosensor monitors lactate non-invasively in human perspiration during exercise	Enables repeated biochemical monitoring during activity rather than relying only on periodic blood testing	47 and 48
Sample collection burden	Paper-based uric acid assays detect uric acid in collected biological samples such as serum or urine	A wireless mouthguard biosensor monitors salivary uric acid using an integrated uricase-modified electrode and bluetooth electronics	Provides a less invasive and more user-friendly route for monitoring gout- or hyperuricemia-related biomarkers	49 and 50
Device format	Conventional point-of-care IL-6 immunosensors are usually electrode- or strip-based platforms used after sample collection	Soft skin-interfaced microfluidic systems can collect sweat on the body and integrate immunoassays, fluorometric sensors, and impedance measurements	Converts biosensing from a separate testing device into a body-conformal monitoring interface	40 and 51
Multiplexed sensing	Nanoplasmonic biosensor microarrays can quantify multiple cytokines from serum under controlled assay conditions	A wearable microfluidic biosensor can monitor multiple stress hormones such as cortisol, epinephrine, and norepinephrine in sweat	Enables simultaneous and dynamic biomarker profiling on the body instead of multiplexing only in a laboratory-style assay	52 and 53
Data interpretation	Conventional electrochemical or colorimetric biosensors usually report a direct signal, concentration, or threshold-based result	An explainable deep-learning-assisted programmable colorimetric sweat chip interprets complex sweat colour responses for automated biochemical assessment	Adds AI-assisted interpretation to optical sweat biosensing, reducing subjective visual reading and improving extraction of meaningful biochemical information from colourimetric signals	51 and 54
Remote healthcare use	Conventional multiplex serum assays provide useful diagnostic data but still require sample collection, processing, and assay operation	A wearable sweat glucose sensing device with electrochemical sweat analysis and machine-learning correlation for instant diabetes diagnosis on a smartphone	Supports smartphone-linked, non-invasive metabolic monitoring and remote screening without repeated clinical sample collection	52 and 55
Patient comfort and compliance	Blood sampling is used in classical enzyme-electrode glucose biosensors as well as in finger-prick glucose systems	Smart contact lenses have been developed for continual glucose monitoring in tear fluid	Improves comfort by shifting from repeated finger-prick blood testing toward less invasive tear-based sensing	56 and 57
Personalized stress monitoring	Salivary cortisol electrochemical biosensors can detect cortisol from collected saliva samples	A wearable aptamer-FET biosensor array can monitor cortisol non-invasively in sweat	Allows stress-related biomarkers to be tracked in a more continuous and personalized manner	39 and 58
Real-world physiological context	Conventional biosensors usually measure one biomarker without capturing body movement, activity state, or surrounding physiological variation	Wearable laser-engraved sensors can combine sweat biomarker sensing with vital-sign monitoring such as temperature and respiration-related measurements	Incorporates physiological context into biochemical data for more appropriate interpretation for monitoring of disease in the real world	38

## 3 Fundamental architecture and working principles of smart wearable biosensors

Smart wearable biosensors function as integrated biochemical-to-digital systems rather than simple miniaturised diagnostic strips. Their performance depends on six connected steps: access to a relevant biofluid, capture of the target analyte, stable immobilisation of the recognition element, conversion of the recognition event into a measurable signal, correction of biological or mechanical interference, and presentation of the output in a clinically interpretable format.^1,59^ Hence, the working principle of a wearable biosensor should be assessed as a continuous signal chain in which any failure at any point in the chain could diminish the reliability of the overall health readout.

### 3.1 Structural components of smart wearable biosensors

The basic components of a smart wearable biosensor are a sampling interface, recognition layer, transducer, electronic readout unit, power source, and display module. However, in wearable systems, these components must operate while exposed to skin deformation, variations in sweat, temperature changes, and motion artefacts. Recent textile-based biosensors illustrate this challenge because the fibre or fabric is not only a support material, but also part of the sensing architecture that must retain conductivity, flexibility, washability, and skin comfort.^60^ Likewise, self-powered sweat sensors reveal that energy management is becoming a component of the device design, particularly if continuous monitoring is needed and batteries are not readily available for frequent replacement.^61^ Printed and 3D-printed biosensors also prove that device architecture can be designed in flexible, stretchable, and anatomically compatible formats, albeit with the concern of batch reproducibility.^62^ Therefore, there is more than miniaturisation to be considered in the design of the structure, it should also carry out stable transportation of analytes, receptor activity, electronic contact and practical wearability.

### 3.2 Biofluid access and sampling pathways in wearable biosensing

The choice of biofluid is a key design factor since the same biomarker might not respond identically in blood, sweat, tears, saliva, interstitial fluids, wound exudate or breath condensate. Sweat is an appealing parameter for non-invasive monitoring because it is present on the surface of the skin, but sweat rate, evaporation, contamination, and mixing within the skin surface can alter the concentration readings.^63^ Some of these issues can be addressed using microfluidic sweat systems, which control fluid routing, separate fresh and old samples, and enable time-resolved analysis.^64^ Interstitial fluid has biochemical similarity to blood and may be accessed by microneedles, but the depth of insertion, extraction efficiency, and tissue response must be carefully controlled.^65^ Tear-based biosensing is a pathway to monitoring the eye and the whole body; however, when considering lens comfort and blinking, tear volume has an impact on signal stability.^66^ The relevance of breath condensate is growing due to the ability to collect and analyse real-time respiratory biomarkers including ammonium, nitrite, pH and alcohol in smart masks.^67^ Wound exudate is clinically useful for wound infection and healing assessment, but the heterogeneity of wounds and variable fluid volumes make quantitative interpretation difficult.^68^ Thus, biofluid access is not just a sampling problem; it is a determinant for the validity of biomarkers, device geometry, calibration requirements, and clinical utility.

### 3.3 Bioreceptor immobilization and interface stability

The recognition layer must be maintained during long-term interaction with complex biofluids. Physical adsorption is easy to perform, but a weak bond may lead to receptor leaching. Entrapment and matrix encapsulation can protect enzymes or probes, although excessive matrix thickness may slow diffusion. Covalent attachment enhances retention, but the wrong coupling chemistry can cause the receptor activity to be decreased. Enzyme-based wearable biosensors reveal that the coupling of enzymes and conductive materials needs to be optimised to maintain enzymatic activity while simultaneously allowing for effective electron transfer.^69^ Other interfaces such as antifouling and hydrogel have been shown to be important as proteins, salts, cells, and wound debris can clog active sites or affect the electrode response.^70^ Molecularly imprinted electrochemical sensors for cortisol reveal that synthetic recognition layers can be made more robust than biological receptors, but need to be tested in real sweat matrices.^71^ A fully printed wound-bandage sensor with pH correction illustrates that interface stability is not merely about holding the receptor in place; it is also about counteracting environmental conditions that can alter the signal.^72^ Another immobilization method gaining traction is bioorthogonal click chemistry, which permits the selective attachment of receptors to the substrate that cannot be bound by nonspecific adsorption or weak self-assembly.^73^ Therefore, the choice of immobilisation strategy should depend on the type of receptor, size of target, complexity of biofluid, diffusion distance and duration of wear.

### 3.4 Recognition chemistry in smart wearable biosensors

The biosensor selectivity is supported by recognition chemistry. However, for metabolites such as glucose, lactate, uric acid, and alcohol, enzymatic reactions can be coupled directly to electrochemical signals, making it possible to use them as suitable indicators.^74^ Antibody-based systems are better for proteins, cytokines, viral antigens and inflammatory biomarkers, but can be costly, difficult to regenerate and less stable during continuous utilization.^75^ Aptamers can reversibly bind their target, are synthetically tunable, and can bind to flexible electrodes, and can therefore be used for hormones, drugs and biomarkers of low abundance.^39^ CRISPR-based recognition is highly specific for nucleic acid targets while the in-wearable translation is difficult due to the challenge of integrating amplification, reagent storage, and sample preparation on the body.^76^ Nanozyme-based systems may have enhanced catalytic stability and signal amplification, but their selectivity tends to be less biomimetic than that of enzymes or antibodies.^77^ MIPs are synthetic binding sites that are stable and inexpensive, particularly for small molecules like cortisol, amino acids, and drugs.^78^ Thus, recognition chemistry should not only be determined by the reported detection limit, but also by the analyte, the biofluid, the transducer, and the anticipated monitoring time.

### 3.5 Selection of recognition elements for wearable applications

The best recognition element for wearable applications is not necessarily the most sensitive one in the lab. Enzymes are potent for rapid metabolite sensing, while they require protective matrices to protect against pH and temperature changes.^79^ Antibodies can offer high specificity for immune and disease proteins, but are not as well-suited for repeated regeneration in the long-term wearable format^80^ Aptamers are promising for reversible and low power sensing when brought together with platforms based on transistors or impedances, though biofluid matrix effects are a limitation.^81^ MIPs are appealing for wearable small-molecule monitoring due to their low cost and chemical stability, while their clinical reliability requires testing against structurally similar interferents.^82^ Functional biological responses can be achieved by cell-based and microbial recognition systems; however, biosafety, viability and long-term control limit the direct wearable application of these systems.^83^ Nanozymes are valuable when natural enzymes are unstable, yet their catalytic response must be separated from nonspecific redox interference.^84^Table 2 provides a comparative overview of recognition chemistry, biofluid compatibility, and translational suitability of wearable biosensor bioreceptors. Therefore, recognition-element selection should be evaluated using wearable-specific criteria such as stability, regeneration, antifouling behaviour, response time, calibration burden, and compatibility with flexible manufacturing.

**Table 2:** Recognition chemistry, biofluid compatibility, and translational suitability of wearable biosensor bioreceptors

Recognition element	Bioreceptor classification	Recognition mode	Biomarkers	Biofluid relevance	Critical advantage for wearable biosensing	Key limitation under wearable conditions	References
Enzymes	Catalytic bioreceptor	Enzyme-catalysed reaction converted into an electrochemical/optical signal	Glucose, lactate, uric acid, alcohol, and cholesterol	Sweat patches, microneedle ISF sensors, tattoo electrodes, textile electrodes	Optimal for rapid metabolite monitoring as catalytic turnover provides rapid signal generation, simple electrochemical coupling	Activity can decrease as pH, temp, drying, preservation, and sweat/ISF exposure is repeated	69
Multienzyme systems	Catalytic bioreceptor	Cascade enzymatic reaction	Cholesterol, glucose-derived H_2_O_2_, complex metabolites	Microfluidic patches and flexible electrochemical platforms where sequential conversion is needed	Useful when one enzyme cannot generate a selective signal alone; cascade reactions can improve analyte specificity	FailOne enzyme failure disrupts the entire cascade; diffusion between layers can delay response	85
Nanozymes	Catalytic bioreceptor	Nanomaterial-based enzyme-like catalysis	H_2_O_2_, lactate-related reactions, oxidative stress markers, infection-related redox markers	Sweat patches, textile biosensors, flexible electrodes, wound sensors	More chemically and thermally stable than natural enzymes; useful when long-term wearable exposure may denature proteins	Lower target specificity and nonspecific redox activity may cause false signals in complex biofluids	86
Antibodies	Non-catalytic affinity bioreceptor	Antigen–antibody binding	CRP, TNF-α, cytokines, troponin, viral antigens, cancer proteins	The new generation of Sweat/ISF immunosensors, microfluidic patches, wound exudate platforms	Best suited for disease proteins that cannot be detected enzymatically; high target specificity supports clinical biomarker detection	Cost, receptor orientation, limited regeneration, and stability loss reduce continuous-use suitability	87
Aptamers	Non-catalytic affinity bioreceptor	Synthetic DNA/RNA target binding	Cortisol, hormones, drugs, proteins, and inflammatory markers	Sweat sensors, ISF sensors, FET platforms, flexible electrochemical electrodes	Smaller and more chemically tunable than antibodies; reversible binding is useful for dynamic biomarker monitoring	Nuclease degradation, nonspecific adsorption, matrix interference, and baseline drift may affect long-term accuracy	3
DNA/RNA probes and CRISPR systems	Non-catalytic/genosensor	Sequence-specific nucleic acid recognition	Viral RNA, miRNA, gene mutations, infectious-disease markers	Smart masks, wearable lab-on-chip systems, microfluidic diagnostic patches	High molecular specificity towards nucleic acid targets in infectious diseases and cancer	Sample preparation, amplification, reagent storage, temperature control and on-body integration are not easy to perform	88
Molecularly imprinted polymers	Synthetic non-catalytic recognition	Template-shaped synthetic binding cavities	Cortisol, drugs, amino acids, hormones, small molecules	Paper microfluidic wearables, flexible electrochemical sensors, sweat patches	Robust, low-cost, reusable, and less fragile than biological receptors; strong option for small-molecule wearable sensing	Cross-reactivity with structurally similar molecules must be tested in real sweat/ISF	82
Receptor-, peptide-, and protein-based recognition	Non-catalytic affinity bioreceptor	Ligand–receptor, peptide–target, or protein–target binding	Hormones, neurotransmitters, metal ions, toxins, microbial markers	Flexible electrodes, optical patches, microfluidic/lab-on-chip wearables	Provides greater flexibility in design compared to full antibodies and can target niche biomarkers	Maturity is limited by degradation, nonspecific adsorption, orientation control, and limited wearable demonstrations	89
Cell-based and microbial systems	Catalytic/living bioreceptor	Whole-cell or microorganism metabolic response	Toxicity markers, metabolic activity indicators, and environmental-biological signals	Mostly experimental hybrid patches or external wearable-assisted modules	Provides functional biological response rather than single molecular binding	Biosafety, viability, nutrient requirement, reproducibility, and regulatory control make direct clinical wearable use difficult	90
Lectin, riboswitch, plant tissue, animal tissue, and organelle systems	Limited wearable relevance group from old catalytic/non-catalytic tables	Glycan binding, RNA conformational recognition, or natural tissue/organelle activity	Glycoproteins, metabolites, ATP, toxicity markers	Mostly conventional biosensors or lab-on-chip formats; weak evidence for body-worn continuous monitoring	Useful historically and for niche biochemical recognition	Lack of reproducibility, lack of stability, poor miniaturisation and low wearability compatibility	91–94

### 3.6 Signal conversion from biochemical recognition to readable wearable output

Once the molecular recognition step is complete, a biochemical event has to be translated into a readable output. The use of electrochemical transduction is widespread because changes in current, potential, impedance or charge transfer can be sensed by low-power, small electronics.^27^ Optical transduction also provides a colorimetric output, a fluorescent output, a plasmonic output, and a camera-readable output, but may be susceptible to ambient light, optical alignment, skin pigmentation and photo instability.^95^ Field-effect transistor and impedance-based systems provide label-free, sensitive readouts, but surface charge screening in high-ionic-strength biofluids can degrade performance.^18^ In the case of microfluidic wearables, signal conversion is tightly coupled with sample routing, as delayed sweat transport or fluid mixing may alter the apparent biomarker response.^96^ Haptic-feedback hydration biosensors show that wearable outputs can move beyond concentration display toward direct user alerts, although such alerts must be carefully validated to avoid overinterpretation.^97^ Smartphone and wireless modules can create accessible output, but only when the sensing chemistry, calibration approach, and biological interpretation are simultaneously validated.^67^ Thus, signal conversion is not the final technical step only; it is the stage where chemical recognition becomes actionable health information.

## 4 Materials chemistry, biointerface design, and transduction pathways in smart wearable biosensors

**Fig. 2:**
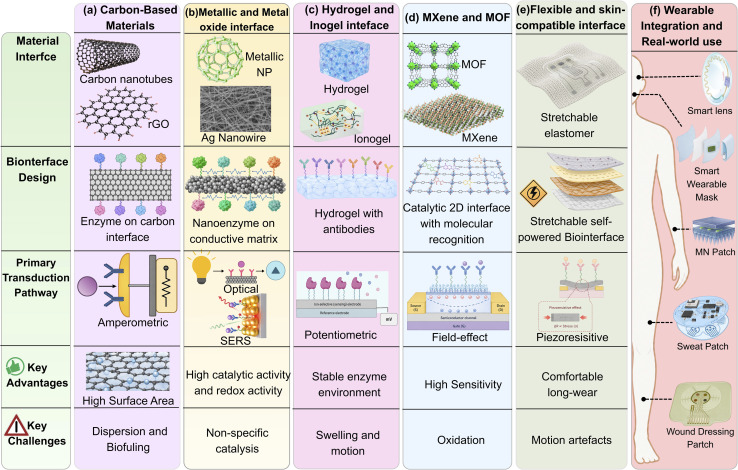
Material interfaces and transduction pathways in smart wearable biosensors. Schematic summary of (a) carbon-based materials, (b) metallic and metal oxide interfaces, (c) hydrogel/ionogel biointerfaces, (d) MXene/MOF platforms, (e) flexible skin-compatible interfaces, and (f) wearable integration formats, highlighting their biointerface design, primary transduction pathways, advantages, and challenges.

The choice of materials for smart wearable biosensors should be considered throughout the entire sensing pathway (Fig. 2). The main concern is the ability of each material to recognize, turn into a measurable signal, and to keep in contact with the skin/biofluid during motion, sweating, dehydration, and extended use. Table 3 summarizes the relationship between material-biointerface design, transduction mechanisms, and wearable performance of smart biosensors.

**Table 3:** Material-biointerface design, transduction modes, and wearable performance of smart biosensors

Material class	Representative smart wearable biosensor	Chemistry/material role	Transduction mode	Sensing mechanism	Flexibility/stretchability	Mechanical strength/fracture behaviour	Adhesion and biointerface compatibility	Mechanical degradation/stability concern	Biodegradability/disposability	References
Graphene and laser-induced graphene	Vertical graphene microneedle biosensor for ISF ketone/glucose monitoring; LIG sweat patch for glucose, lactate, and sodium	High electroactive surface area, conductive edge-plane sites, porous graphene network, enzyme-supporting surface	Electrochemical; amperometric; potentiometric	Enzymatic glucose/ketone/lactate reactions generate current; ion-selective layers produce potential changes	High flexibility in LIG patches; microneedle format improves ISF access	Vertical graphene microneedles require adequate fracture resistance for skin insertion	Good epidermal or ISF contact, but adhesion and irritation must be controlled	Signal may be affected by graphene fouling, sweat evaporation, microneedle fracture and motion artefacts	Not biodegradable; disposable patch or microneedle formats must be removed safely	126 and 127
rGO, CNTs, and carbon fibres	rGO sensing yarn for continuous sweat lactate monitoring; CNT-enzyme polymer sweat biosensor	Conductive fibrous network, high aspect ratio, enzyme-supporting scaffold, improved electron transfer	Amperometric; voltammetric; impedimetric	Enzyme-catalysed sweat biomarker reactions generate current or impedance changes	Strong textile compatibility; suitable for yarns, fibres, and clothing integration	Carbon fibres and rGO yarns are mechanically robust but may deform under repeated bending or washing	Comfortable in textile form, but skin–fabric friction and sweat distribution affect accuracy	Washing, bending, CNT dispersion, possible cytotoxicity, and nonspecific adsorption are concerns	Not readily biodegradable; may contribute to disposal problems if integrated into textiles	102
Metal, metal oxide, and nanozyme composites	MWCNT–iron oxide printed sweat glucose sensor; nanozyme–enzyme sweat lactate sensor	Catalytic amplification, redox activity, H_2_O_2_ conversion, improved electron transfer	Electrochemical; mainly amperometric	Glucose or lactate reactions are converted into current through catalytic nanomaterial/enzyme interfaces	Compatible with flexible printed electrodes and textile-assisted sweat collection	Mechanical strength depends on ink adhesion and nanocomposite retention on flexible substrate	Can enhance sensitivity, but metal/oxide particles need stable encapsulation for skin contact	Drift may occur due to nanoparticle aggregation, leaching, oxidation, non-specific catalysis and textile fouling	Poor biodegradability; devices with nanoparticles need safe disposal	104 and 128
Plasmonic and SERS-active biopolymer systems	Silk fibroin-based wearable SERS biosensor for sweat creatinine and uric acid; SERS-AI sweat platform for gout diagnosis	Biocompatible silk matrix supports sweat absorption; plasmonic nanostructures amplify Raman signal	Optical; SERS-based	Molecular vibration fingerprints of sweat biomarkers are enhanced by plasmonic hot spots	Flexible and skin-compatible when integrated with silk or soft substrates	Mechanical stability depends on plasmonic substrate integrity and silk film durability	Silk improves comfort and biocompatibility, but stable sweat contact is still needed	Reproducibility is affected by Raman signal variability, hotspot instability, sweat evaporation, and fouling	Silk is more biodegradable than synthetic polymers, but plasmonic nanoparticles are not	129
Conductive polymers and hydrogels	PEDOT : PSS composite hydrogel microneedle electrode; zwitterionic hydrogel enzyme biosensor; self-healing hydrogel sweat sensor	Mixed ionic–electronic conductivity, hydrated polymer network, enzyme stabilisation, antifouling hydration layer	Amperometric; potentiometric; impedimetric	Biomarker concentration changes current, potential, or impedance through hydrated conductive interface	High softness and stretchability; reduces mismatch between tissue and electronics	Hydrogels tolerate deformation but may tear, swell, or lose shape under strain	Strong biointerface advantage due to softness, hydration, and possible adhesion	Dryness, swelling, hysteresis, poor bonding, enzyme leakage, and fatigue are all problems with long-term use	Some hydrogels are partially biodegradable; synthetic conductive polymers are less degradable	130
MXenes, MOFs, COFs, and BP/g-CN heterostructures	MXene Bio-FET sweat glucose patch; MOF-derived porous carbon sweat sensor; BP/g-CN NFC sweat glucose patch	High surface area, hydrophilic terminations, porous catalytic sites, improved charge transfer and analyte adsorption	FET-based; electrochemical; non-enzymatic	Sweat biomarkers modulate transistor current or undergo oxidation/reduction at catalytic surfaces	Flexible when combined with paper, PDMS, hydrogels, or microfluidic skin patches	Mechanical strength depends on composite design; 2D layers may crack or delaminate	Good sensitivity, but interface needs encapsulation and calibration against sweat variation	MXene oxidation, MOF poor conductivity, pore fouling, BP oxidation, and sweat interference are key issues	Generally limited biodegradability; device disposal will depend on substrate and nanomaterial loading	116 and 131
Flexible substrates: Paper, textile, elastomer, tattoo-like, silk, collagen, and microneedles	Paper Bio-FET patch, textile sweat sensor, tattoo-like electrochemical patch, silk/collagen biointerface, microneedle ISF device	Provides conformal contact, sweat/ISF access, fluid routing, mechanical support, and user comfort	Depends on integrated sensor: Electrochemical, optical, FET, colorimetric, or SERS	Substrate controls biofluid sampling, analyte diffusion, electrode deformation, and receptor stability	Paper is bendable; textiles are wearable; elastomers are stretchable; microneedles provide ISF access	Microneedles require insertion strength; tattoos and elastomers require resistance to cracking	Determines skin comfort, adhesion, irritation, breathability, and sampling reliability	Drying, delamination, washing damage, skin irritation, substrate swelling, and fracture may occur	Paper, silk, and collagen are more biodegradable; elastomers and tattoos are less biodegradable	48 and 132
Self-powered and bio-derived biointerfaces	Fingertip sweat-powered microgrid; microbial-derived bio-hydrovoltaic e-skin; fully organic self-powered e-skin	Sweat biofuel cells, moisture-driven charge generation, triboelectric layers, bio-derived porous structures	Electrochemical energy harvesting; bio-hydrovoltaic; triboelectric	Sweat or moisture generates electrical output and may power integrated metabolic sensors	Designed for flexible, body-conformal and battery-free operation	Mechanical robustness depends on repeated bending, friction, and encapsulation	Reduces external battery dependence; skin comfort and breathability remain important	Power fluctuation, moisture dependence, delamination, encapsulation failure, and integration with biochemical recognition are challenges	Electronics and electrodes remain a barrier to full biodegradability despite bio-derived components being part of the picture	123,133 and 134

### 4.1 Carbon-based materials in smart wearable biosensors

Carbon materials provide conductive, flexible, and chemically modifiable interfaces when electrochemical sensitivity must be combined with compliance, textile integration, or epidermal contact.

#### 4.1.1 Graphene and laser-induced graphene interfaces

A vertical graphene microneedle biosensor could provide real-time monitoring of ketones and glucose during ketogenic diet management by accessing interstitial fluid with a minimally invasive array.^98^ Chemically, vertical graphene offers a high electroactive surface area, edge-plane conductive sites, and fast electron-transfer pathways for enzyme-supported electrochemical sensing.^98,99^ Transduction is primarily amperometric, which involves enzymatic reactions being converted into current responses related to glucose and ketone. The performance, however, relies on the strength of the microneedles, depth of penetration, skin irritation, adhesion, and motion stability.^98^

Sweat glucose, lactate and sodium monitoring has been achieved using laser-induced graphene in conjunction with polymeric tape microfluidics. The laser carbonisation process generates porous, defect-rich electrodes. Glucose and lactate are measured amperometrically and sodium is measured potentiometrically. Microfluidics improves sweat routing, but evaporation, fouling, motion artefacts, and adhesive failure remain limitations.^100,101^

#### 4.1.2 Reduced graphene oxide, carbon nanotube, and carbon nanofiber interfaces

The development of the reduced graphene oxide yarns has made it possible to monitor the sweat lactate level continuously and wirelessly from the textiles. Lactate oxidase and mediator layers are supported by rGO fibres as a conductive and functionalizable electrode. Lactate detection is amperometric, while pH correction improves interpretation. The advantage is comfort, but the washing durability, friction between skin and fabric, uneven sweat distribution and skin fibre deformation may impact the reproducibility.^102^

CNT-induced enzyme polymerisation-based biosensors have been reported for the detection of sweat glucose, uric acid, and pH. CNTs deliver conductive nanoscaffolds. The mechanism is electrochemical since enzyme reactions produce current responses proportional to biomarkers. However, dispersion difficulty, possible cytotoxicity, nonspecific adsorption, and weak adhesion require coatings, encapsulation, or hydrogel interfaces.^103^

Carbon nanofibers and carbon composites offer porous conductive networks for enzyme loading and electrolyte diffusion, but their transduction is typically amperometric, voltammetric, or impedimetric, and they are subjected to bending, washing, fatigue, and biofouling that limit textile translation.^102,103^

### 4.2 Metallic, metal oxide, and plasmonic nanomaterial interfaces

Metallic and metal oxide nanomaterials add electrocatalytic, redox, and optical-amplification functions, but aggregation, leaching, nonspecific catalysis, and biofluid instability require composite design and protective interfaces.^100^

#### 4.2.1 Electrocatalytic metal and metal oxide interfaces

An all-printed wearable biosensor based on MWCNT-iron oxide nanocomposite ink was reported for sweat glucose detection. The MWCNTs enhanced the conductivity and the iron oxide added the catalytic activity. Current changes were used to quantify sweat glucose, but aggregation, detachment, and non-specific catalytic responses must be overcome by stable inks and protection coatings.^104^

A nanozyme–enzyme wearable electrochemical biosensor using CeO_2_–MoS_2_ nanozyme, Au nanoparticles, and a Janus textile sweat-transport layer enabled sweat lactate monitoring. Lactate oxidase generated hydrogen peroxide and nanozyme/Au interface increased reduction, making the signal amperometric. The Janus textile enhanced sweat flow, but enzyme denaturation, fouling, evaporation, and deformation may impact stability.^86^

The flexible biosensors have also been applied in the non-enzymatic detection of sweat glucose by introducing Pt as catalytic oxidation sites and Ti_3_C_2_T_*x*_ MXene for enhancing the conductivity and hydrophilic interaction with sweat. Although enzyme instability is avoided, sweat interferents, MXene oxidation, delamination, and unstable skin contact remain concerns.^105^

A silver nanowire-loaded electrothermal patch improved sweat availability for continuous sweat glucose analysis at rest. The release of joule heat by the percolation network of silver nanowires causes local sweating, while the electrochemical nature of glucose recognition remains unchanged. Therefore, it is a thermal-assisted electrochemical biosensor, not just a calorimetric sensor. Some major concerns include skin-safe heating, temperature uniformity, comfort, evaporation control, adhesive stability and thermal-motion artefacts.^106^

#### 4.2.2 Plasmonic and SERS-active optical interfaces

Plasmonic and SERS-active materials enable optical molecular fingerprinting. A silk fibroin-based wearable SERS biosensor monitored sweat creatinine and uric acid simultaneously. Silk fibroin offered a flexible biocompatible matrix that absorbed sweat, and plasmonic nanostructures served to enhance the Raman signals. The interface benefit is comfort, whereas reproducible sampling, substrate stability, and Raman variation remain limitations.^107^

An intelligent sweat platform that combines SERS and AI was also reported for the diagnosis of gout, in which Raman-enhanced sweat profiles were classified by AI-based methods.^108^ For translation, controlled sweat collection, stable hot spots, antifouling surfaces, and clinically validated datasets are required. The non-electrochemical sweat sensors that have been investigated, such as colorimetric, fluorescence, SERS, and plasmonic sensors, are remain vulnerable to light exposure, evaporation, and imaging conditions.^109^

### 4.3 Conductive polymer, hydrogel, ionogel, and organohydrogel biointerfaces

The mismatch between electronics and soft tissues is minimized using conductive polymers and hydrogels. The composite hydrogels of PEDOT : PSS are proposed as a flexible electrode for wearable microneedle sensing platforms. PEDOT : PSS is a mixed ionic–electronic conductive, and the hydrogel is hydrating and diffusing the analytes, with the transduction being amperometric, potentiometric, or impedimetric. Long-term accuracy can be affected by swelling and dehydration, weak adhesion, and hysteresis.^110^

The zwitterionic hydrogels are able to preserve the oxidase stability because they form hydration layers, prevent nonspecific adsorption, and preserve the enzyme conformation, enabling enzyme-mediated electrochemical sensing with better antifouling properties. Durability may be limited by excessive swelling, slow diffusion, and weak mechanical strength.^111^

Antifouling and self-healing hydrogel sweat sensors minimize biofouling and restore mechanical/electrical continuity *via* polypeptide networks and dynamic bonds. They enhance their electrochemical output when bent, but they must still be breathable to be adherent and easy to remove without causing discomfort.^112^

Hydrogel dehydration is overcome using ionogels and organohydrogels. Ionic pathways remain in poly(ionic liquid) ionogels, which may be resistive, capacitive, or biopotential readouts, and whose biochemical relevance relies on integration with recognition chemistries.^113^ Anti-drying behaviour, elasticity and adhesion to skin are obtained with self-adhesive biocompatible organohydrogels, but analyte diffusion and long-term biocompatibility should be validated.^114^

A wireless parity-time-symmetric bioresonator detected biological signals such as tear glucose and blood lactate using resonant readout. The electrical or mechanical response changes after interacting with glucose or lactate in the case of the biomarker-responsive recognition components, with the resonator amplifying these changes as resonance shifts. This provides high sensitivity and wireless detection, but translation requires stable biofluid contact, encapsulation, temperature calibration, and resistance to motion artefacts.^115^

### 4.4 MXene, MOF, COF, and emerging two-dimensional material interfaces

MXenes, MOFs, COFs, and 2D heterostructures offer high surface area, tunable chemistry, porosity, and catalytic activity. An MXene-enhanced organic Bio-FET paper patch was reported for sweat glucose detection with pH and temperature calibration. A conductive channel formed by Ti_3_C_2_ MXene, CuO, and MWCNT-COOH, with calibration elements to compensate for sweat fluctuation. The mechanism was field-effect transduction, in which biochemical changes affected channel current. The challenges include pH/temperature calibration, paper wetting instability, and MXene oxidation.^116^

MOF-derived porous carbon has been used in microfluidic wearable electrochemical sensors for sweat metabolites and electrolytes. Porous architecture and active sites enhance analyte diffusion and electrochemical response, and microfluidics enhance sampling capability. But pore fouling, variation in sweat rate and contact instability are still limiting factors.^117^

A Ni–Co MOF nanosheet-coated Au/PDMS sensor enabled continuous sweat glucose monitoring *via* non-enzymatic glucose oxidation at electrocatalytic MOF sites. Au/PDMS provides flexibility and high conductivity, but adhesion, catalytic drift, and sweat salts should be controlled.^118^

Black phosphorus/graphitic carbon nitride heterostructures have also been used in NFC microfluidic skin patches for real-time sweat glucose monitoring. Phosphorus–nitrogen interactions increase the electrochemical surface area, decrease charge-transfer resistance and increase glucose adsorption, while BP oxidation, sweat interference and stability in encapsulation are important issues.^79^

Materials with piezoelectric and acoustic-wave properties are emerging interfaces for label-free mass, viscosity, density, or binding-induced changes. Surface acoustic wave biosensors are based on piezoelectric substrates and interdigital transducers that detect biochemical or interfacial changes as changes in wave velocity, amplitude, phase, or frequency.^119^ In wearable formats, the surface must be functionalised with antibodies, aptamers, enzymes, or molecular recognition layers. This approach is less mature than electrochemical sweat sensing because bending strain, temperature fluctuation, sweat viscosity, and imperfect contact can distort acoustic signals. Antifouling coatings, flexible encapsulation, and mechanically stable piezoelectric substrates are therefore essential.^120^

### 4.5 Flexible, skin-compatible, and self-powered biointerfaces

A microgrid integrated into a handheld device was shown to autonomously manage energy and monitor metabolic data during activity, sweating and extended wear. The enzymes were used in biofuel cells to utilise energy from sweat, and sensors were integrated to detect glucose, vitamin C, lactate, and levodopa. The mechanism was electrochemical and energy integrated since the sweat served as an analytical biofluid and energy source.^121^ Stable adhesion, encapsulation, breathability, and resistance to motion artefacts remain necessary for long-term use.

Under flexible substrates, the issue of mechanical transduction is reported, as flexible wearable biochemical sensors are constantly bent, stretched, and compressed. Strain-tolerant biointerfaces can be used in combination with biochemical sensing and include conductive polymer hydrogels, elastomers, organohydrogels, and textile composites. The chemical composition of these materials consists of hydrated polymer networks, ionic pathways, dynamic bond networks, or conductive fillers that ensure signal transport even during deformation. Their readouts may be resistive, capacitive, piezoresistive, or impedance-based, while biochemical layers detect sweat or interstitial-fluid biomarkers. Key challenge is the separation of true biomarker variation from mechanical artefact, requiring motion correction, antifouling surfaces, breathable encapsulation, and removable adhesion.^122^

Bio-derived self-powered interfaces are gaining attention. A microbial-derived bio-hydrovoltaic electronic skin showed moisture-responsive self-powered multisensory sensing through porous bio-derived ion/electron transport.^123^ Although not yet a direct biochemical biosensor, it is identified as a battery-free interface design. Collagen-derived electronic skins are relevant since collagen is biocompatible and biodegradable, has a skin-like mechanics, and offers bioactive sites that could support tissue-compatible biosensing.^124^ Fully organic triboelectric electronic skin further shows how soft materials can generate sensing signals without external power.^125^

## 5 Platform-specific applications of smart wearable biosensors in disease diagnosis and therapeutic monitoring

**Fig. 3:**
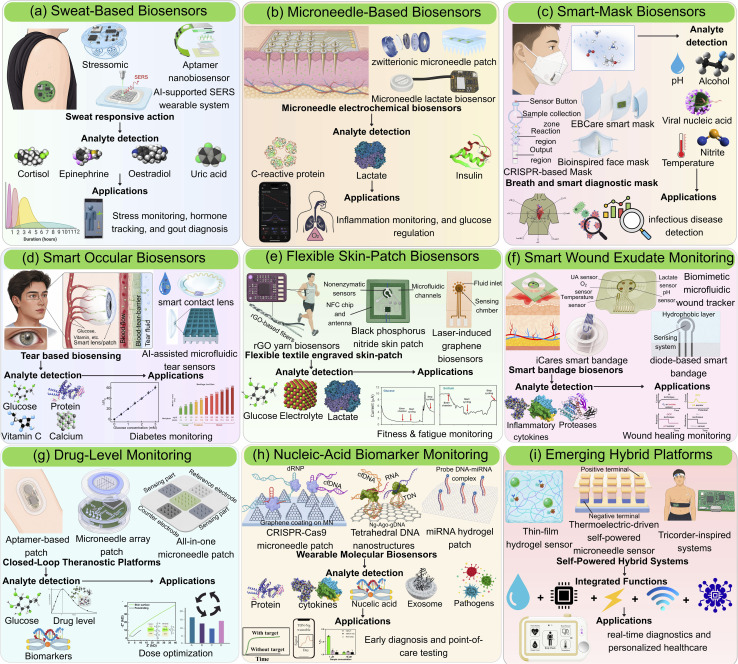
Platform-specific applications of smart wearable biosensors in disease diagnosis and therapeutic monitoring. (a) Sweat-based biosensors for monitoring stress, hormonal and metabolic biomarkers, (b) microneedle-based biosensors for interstitial-fluid detection of inflammatory, metabolic and protein biomarkers, (c) smart-mask biosensors for exhaled breath condensate and respiratory aerosol analysis, (d) smart ocular biosensors for tear-based monitoring of glucose, proteins, ions and ocular/systemic health markers, (e) flexible skin-patch and textile biosensors for sweat-based fitness, fatigue, hydration and metabolic monitoring, (f) smart wound-exudate biosensors for tracking oxygen, pH, temperature, lactate and inflammatory mediators during wound healing and infection, (g) drug-level monitoring and closed-loop theranostic platforms for dose optimisation and personalised therapy, (h) wearable molecular biosensors for nucleic-acid, cytokine, exosome and pathogen detection, (i) emerging hybrid platforms integrating optical, self-powered, wireless and multimodal sensing functions for real-time diagnostics and personalised healthcare.

Smart wearable biosensors are valuable because they enable continuous and real-time monitoring of physiological and biochemical parameters. Unlike conventional laboratory testing, they allow body-integrated assessment of biomarkers over time. These devices can analyze various biofluids, including sweat, saliva, tears, interstitial fluid, and wound exudate. Their importance lies not only in biomarker detection but also in tracking dynamic changes in health status. Their applications can be understood through wearable platform design, target biofluid, sensing mechanism, and clinical relevance (Fig. 3). An application is presented in the context of the biofluid, biomarker, recognition chemistry, transduction mechanism, clinical relevance and key platform-specific constraints.

### 5.1 Sweat-based biosensors

Wearable biosensors have demonstrated great promise in the field of non-invasive health monitoring *via* sweat analysis. Stressomic, a microfluidic sweat biosensor, includes gold nanodendrite decorated laser-engraved graphene electrodes and hormone-selective electrochemical interfaces to detect cortisol, epinephrine, and norepinephrine for dynamic stress profiling. It offers applications in stress physiology, mental health monitoring, and neuroendocrine assessment, although factors such as sweat rate and hydration influence accuracy.^53^

A wearable aptamer nanobiosensor has been designed for monitoring female hormones by detecting oestradiol in sweat *via* aptamer-based strand displacement and wireless electrochemical sensing. It is also beneficial for tracking fertility, menstrual health, and menopause, and requires reliable low-concentration detection and sweat–blood correlation.^135^

Similarly, an AI-supported SERS wearable system has been developed to detect uric acid in sweat through plasmonic nanostructures, Raman signal enhancement, and neural-network analysis, with the need for additional clinical testing.^108^

### 5.2 Microneedle-based biosensors

Microneedle electrochemical biosensors are valuable because they access interstitial fluid, providing clinically relevant information for systemic biomarker monitoring. Recent research has emphasized the importance of optimized microneedle design, selective recognition chemistry, electrochemical sensing, and comprehensive *in vitro*, *ex vivo*, and *in vivo* validation for successful clinical translation.^136^

A microneedle-based biosensor for the interstitial fluid (ISF) has been designed for the detection of C-reactive protein (CRP), allowing for the monitoring of inflammation by means of affinity-based recognition and electrochemical impedance measurement. It has promising clinical application, but antibody stability, extraction efficiency of ISF, and the validation of the diagnostic thresholds remain challenges.^137^

Wearable microneedle (MN) sensors for continuous ISF monitoring are developing as revolutionary instruments for real-time biochemical analysis in healthcare. In contrast to point-of-care (POC) devices that yield results at a single time point, continuous MN systems offer time-resolved molecular data across prolonged durations, facilitating the identification of swift physiological alterations and aiding in prompt, tailored therapies.^138^

A zwitterionic microneedle patch for continuous insulin monitoring has been developed by incorporating antibody-protected electrochemical sensing for dynamic *in vivo* protein detection, and overcoming protein fouling and sensor stability concerns in protein biomarker microneedle biosensing.^139^

### 5.3 Breath and smart-mask biosensors

The EBCare smart mask is a wearable biosensor with electrochemical sensing for exhaled breath condensate that outputs data wirelessly to a smartphone, measuring pH, alcohol, ammonium, nitrite, and temperature. It is valuable for respiratory inflammation, asthma/COPD, and metabolic monitoring, with performance influenced by condensation efficiency and humidity calibration.^67^

A bioinspired face-mask system allows for real-time measurement of metabolic and respiratory biomarkers in compressed breath through nanoengineered sensing layers that achieve optical or electrochemical transduction. It has potential applications in respiratory health care and exposure monitoring, though its sensitivity is influenced by airflow variability and reproducible collection of condensates.^140^

The diagnostic face mask integrates a CRISPR-based assay to detect viral nucleic acids from the respiratory aerosol with colorimetric or fluorescent output that remains freeze-dried. This platform enables fast screening for infectious diseases, and reagent stability, controlling activation, and multiplex pathogen detection are crucial for practical translation.^46^

### 5.4 Tear-based smart contact lens and eye-patch biosensors

A wireless soft smart contact lens has been developed for continuous tear glucose monitoring, using glucose-responsive sensing with wireless electrochemical/optical signal readout. It is a non-invasive method for diabetes monitoring, and its performance is affected by blinking, tear dynamics, lens comfort, and the correlation between the blood glucose level and tear level.^66^

The plasmonic smart contact lens allows glucose sensing in tears with the use of glucose-responsive optical materials and plasmonic signal transduction. This platform is promising for electronics-free ocular sensing, with reliability depending on optical stability, tear dilution, and ocular biocompatibility.^33^

An AI-assisted microfluidic colorimetric wearable sensor monitors tear biomarkers such as vitamin C, pH, calcium, and proteins *via* reagent-based colorimetric detection and smartphone-assisted image analysis. It can aid in monitoring ocular and systemic health, and the accuracy is dependent on the lighting conditions, the control of tear volume, and the variability of the camera.^141^

### 5.5 Flexible epidermal, textile, and skin-patch biosensors

To enable continuous monitoring of sweat lactate, reduced graphene oxide (rGO) yarn biosensors are developed through an amperometric electrochemical sensing platform on which the lactate oxidase enzyme is immobilized on conductive fibres. They are good for sports performance and fatigue monitoring; they are effective depending on the durability of the wash, the friction of the textiles and sweat distribution.^102^

A black phosphorus/graphitic carbon nitride skin patch, which uses a non-enzymatic phosphorus–nitrogen heterostructure chemistry with electrochemical signal transduction, can allow real-time sweat glucose monitoring. This provides an enzyme-free metabolic sensing method in which the black phosphorus oxidation and sweat interference affect the stability of the sensor.^79^

A combination of enzyme- and ion-selective recognition and amperometric and potentiometric sensing has been integrated into laser-induced graphene epidermal biosensors used to monitor glucose, lactate, and sodium in sweat. These systems are designed to monitor metabolism and hydration; their performance relies on sweat evaporation control, skin adhesion and motion artefact correction.^100^

### 5.6 Smart wound bandages for exudate-based monitoring

The development of a biomimetic microfluidic wound tracker to continuously monitor oxygen in wound exudate, where oxygen-responsive sensing is integrated into microfluidics to evaluate wound healing and tissue oxygenation. It is dependent on exudate variability, dressing changes and long-term skin retention.^142^

The iCares smart bandage is a wearable microfluidic dressing equipped with flexible nanoengineered sensor arrays using electrochemical sensing to detect NO, H_2_O_2_, O_2_, pH, and temperature biomarkers to monitor wound exudate. It is highly relevant to diabetic wound management and infection monitoring and is utilized in practice for scalable fabrication and interpretation of complex wound biochemistry.^143^

A liquid diode-based smart bandage enables monitoring of wound pH using a 3D polyaniline mesh biosensor with potentiometric transduction. This system enables chronic wound management and assessment of the healing phase, providing enhanced infection discrimination and diagnostic accuracy when used in conjunction with other biomarkers.^68^

### 5.7 Drug-level monitoring and closed-loop theranostic platforms

A wearable electrochemical aptamer-based patch has been advanced into pilot clinical trials for the continuous and dynamic monitoring of therapeutic drugs, such as vancomycin, through reversible aptamer-drug binding and electrochemical transduction. It has high precision dosing potential, but is affected by patient variation, aptamer regeneration, and drug-specific validation.^144^

For the continuous monitoring of methotrexate, a microneedle array patch has been developed using voltammetric transduction with a drug-selective electrochemical sensor to measure drug concentration. The system is useful in preventing toxicity and optimizing chemotherapy or anti-inflammatory treatment, and clinical application requires an accurate ISF–blood pharmacokinetic correlation.^145^

An autonomous all-in-one microneedle patch has been reported for closed-loop melanoma diagnosis and therapy, integrating responsive microneedle chemistry with sensing-guided therapeutic actuation. It is a sophisticated theranostic platform for cancer monitoring, where safety, dosimetry control, and tumour-specific validation are crucial to facilitate clinical translation.^146^

### 5.8 Wearable molecular biosensors for nucleic-acid biomarker monitoring

A programmable CRISPR-Cas9 microneedle patch has been developed for real-time monitoring of cell-free DNA (cfDNA) in interstitial fluid, using CRISPR-activated graphene biointerfaces with electrochemical sensing. It demonstrates promising potential in various applications such as viral detection, sepsis, transplant monitoring, and cancer liquid biopsy, where mutation specificity, anti-interference capability, and clinical validation play roles in its performance.^147^

Fully integrated wearable electronics based on tetrahedral DNA nanostructures and prokaryotic Argonaute technology enable ultratrace nucleic acid monitoring through programmable molecular recognition with electrochemical and wireless signal transduction. This platform holds potential for the detection of infectious diseases and molecular surveillance, but it needs to be validated in real patient biofluids.^148^

A hydrogel microneedle patch has been developed for the rapid collection of miRNAs from interstitial fluid, using nucleic acid hybridization with potential downstream molecular or electrochemical readout. It has value in cancer prognosis and post-treatment monitoring, and ongoing efforts are underway for continuous wearable integration.^149^

### 5.9 Emerging image-readable, self-powered, and hybrid platforms

Using metabolite-responsive optical microdroplet chemistry and wavelength-multiplexed laser transduction, a wearable thin-film hydrogel laser has been developed for monitoring metabolites in sweat, including glucose, lactate, and urea. It is an advanced photonic biosensing platform whose performance is strongly dependent on the stability of the optics, adhesion to the skin, and calibration in varying sweating conditions.^150^

Continuous interstitial fluid glucose monitoring is achieved with glucose oxidase-based electrochemical sensing and a skin-conformable thermoelectric generator that enables a thermoelectric-driven self-powered microneedle sensor. This provides biochemical monitoring with reduced battery power, and the efficiency depends on the temperature gradients, the power storage, and long-term biocompatibility.^151^

Tricorder-inspired systems, such as the Berkeley Tricorder, represent hybrid health-monitoring platforms that track physiological parameters including ECG, EMG, respiration, oxygenation, and motion. Although not a classical biosensor, their significance lies in the future integration of biochemical sensing with wireless, AI-assisted, and self-powered wearable monitoring systems.^152^

## 6 AI-assisted signal processing in smart wearable biosensors

AI-assisted signal processing is becoming important in smart wearable biosensors because on-body biochemical signals are affected by motion, lighting, biofluid dilution, temperature, pH, electrode drift, and biofouling. Unlike conventional biosensors, wearable biosensors continuously generate time-dependent and multidimensional data. AI can thus be useful when it enhances signal correction, calibration, classification, multimodal fusion and predictive decision support with measurable performance output.

### 6.1 Noise reduction and signal correction

AI is particularly beneficial in wearable colorimetric biosensors due to the high sensitivity of optical readings to light, camera angle, and ambient color. In the AI-WMCS system, a flexible PDMS microfluidic tear patch was used to collect human tears and detect vitamin C, H^+^/pH, Ca^2+^, and proteins. A colorimetric recognition chemistry was used, and errors arising from the pH and environmental colour-temperature variation were corrected using a multichannel CNN-GRU model. The AI model was found to be accurate with only approximately 20 µL of tear fluid, achieving *R*^2^ values of 0.998 for predicting pH and 0.994 for the other three biomarkers.^141^ This directly supports the need for signal correction assisted by AI, as the model has been found to enhance the accuracy of smartphone-based colour interpretation.

Cotton-textile wearable colorimetric sweat sensor for pH and glucose monitoring. This platform utilized sweat as the biofluid, pH and glucose as biomarkers, and smartphone image capture for analysis. Linear discriminant analysis demonstrated the highest average accuracy of 90.5% (4.3% SD) across fivefold cross-validation for pH detection. For glucose detection, SVM achieved 95.1% accuracy for the TMB-based glucose sensor and 90.0% accuracy for the KI-based glucose sensor.^153^ These results show that AI can convert simple colourimetric sweat patches into more quantitative wearable biosensors.

### 6.2 Sensor drift compensation and calibration

Sensor drift is a major challenge in continuous wearable biosensors because long-term contact with skin or interstitial fluid can change electrode response. In the electronic multiplexed microneedle-based biosensor patch, eMPatch, modular microneedle sensors monitored glucose, uric acid, cholesterol, sodium, potassium, and pH in interstitial fluid. The platform demonstrated analytical stability with less than 2.64% RSD over 14 days of storage and less than 2.21% RSD for batch-to-batch repeatability. When combined with a multi-task convolutional neural network, the system achieved 0.996 classification accuracy for distinguishing normal and diet-induced metabolic disorder and an *R*^2^ of 0.977 for evaluating metabolic disorder degree. This demonstrates evidence of strong AI-assisted calibration and interpretation, as the algorithm transformed drifting, high-dimensional electrochemical data to stable health-state prediction.^154^

A self-calibrating multiplexed microneedle electrode array, SC-MMNEA, further supports the importance of calibration in wearable biosensors. This system monitored glucose, cholesterol, uric acid, lactate, ROS, Na^+^, K^+^, Ca^2+^, and pH in subcutaneous interstitial fluid. Although this study is mainly a self-calibration hardware strategy rather than a pure AI model. The study demonstrated improved reliability after self-calibration, especially for long-term enzyme-based sensing affected by tissue variation and enzyme degradation.^155^

### 6.3 Pattern recognition and anomaly detection

When wearable biosensors produce complex spectra instead of concentration readings, AI based pattern recognition can be useful. A hydrogel-based flexible wearable sweat sensor combined SERS with machine learning for monitoring the treatment effect of lung cancer. The wearable platform used sweat as the biofluid and SERS spectra as the biochemical signal. Machine-learning models including LightGBM, Gaussian Naive Bayes, linear discriminant analysis, random forest, and SVM, were trained on SERS features. The model achieved 89.7% accuracy for lung cancer progression classification.^156^

AgNW/MXene wearable sweat sensor for non-invasive SERS-based cardiovascular disease detection uses sweat cholesterol as the biomarker and AgNW/MXene hydroxyl composite membranes as the SERS substrate. The system detected cholesterol down to 10^−8^ M and maintained performance over 50 stretch release cycles.^157^ Cardiovascular-disease-related sweat profiles were classified by machine learning. This supports anomaly detection as AI can help distinguish disease-associated spectral changes from mechanical deformation or signal fluctuation.

### 6.4 Multimodal biosensor data fusion

Multimodal fusion is important because one biomarker rarely explains the complete physiological state. Multimodal biosensor fusion using AI boosts wearable sensing by addressing biochemical signal overlap and individual variations. An eMoS_*x*_-LIG multimodal electrochemical biosensor platform for sweat and saliva simultaneously detected tyrosine and uric acid, improving detection limits 100-fold to 100 nM and 10 nM, respectively. On-body validation indicated potential for accurate multiplexed, on-body monitoring, but wider clinical validation remains necessary.^158^

The all-flexible chronoepifluidic nanoplasmonic patch, CEP-SERS patch, is another platform. It gathers consecutive sweat samples and employs label-free SERS to profile lactate, uric acid, and tyrosine. The machine-learned quantification was found to be in good agreement with reference assays, with *R*^2^ values of 0.96 for uric acid and 0.86 for lactate, and the error was approximately 5 µM for the prediction of tyrosine.^159^ This demonstrates AI-assisted fusion of time-resolved sweat sampling, multiplexed SERS spectra, and metabolic profiling.

### 6.5 Predictive alerts and clinical decision support

Predictive clinical decision support is most effective when wearable biosensor data are linked to clinically meaningful outcomes. Machine learning models using continuous glucose monitoring (CGM) data predicted metabolic subphenotypes of Type 2 Diabetes, including muscle insulin resistance and β-cell deficiency, with AUC values ranging from 84–95%, enabling precision metabolic assessment.^160^ Similarly, the Accu-Chek SmartGuide Predict App used an XGBoost model to predict hypoglycemic events 30 minutes in advance, achieving 87.13% sensitivity, 97.43% specificity, and ROC-AUC 0.9787. There is a need for comparable clinical validation of emerging sweat, tear, wound, and microneedle biosensors.^161^ Overall, AI should be presented as a validated support layer that improves measurable biosensor performance through correction, calibration, classification, multimodal interpretation, and predictive alerts.

## 7 Translational readiness, clinical validation, and real-world challenges of smart wearable biosensors

A crucial obstacle for smart wearable biosensors is their clinical translation, as they need to be operated outside a laboratory setting and provide clinically relevant biochemical data. These differences are caused by biofouling, temperature/pH changes, user manipulation, breath instability, skin motion, sweat dilution, wound-exudate heterogeneity, and tear dilution, which differ from benchtop assays. Translation therefore requires stable biofluid access, selective recognition chemistry, reproducible transduction, biocompatible integration, secure connectivity, and clinically validated algorithms.

### 7.1 Comparison with conventional laboratory and point-of-care biosensors

A controlled sampling, calibrated instrumentation, trained staff and internal quality control make conventional laboratory biosensors the gold standard in the clinical field, due to the precise determination of biomarkers. Point-of-care biosensors reduce turnaround time but still depend on discrete sampling. ISO 15197:2013 requires at least 95% of blood glucose meter results to fall within strict accuracy limits, illustrating the validation standards expected for clinical biosensors.^162^

Smart wearable biosensors vary in their ability to record dynamic changes in biomarkers, early warning signs, and patterns of response to treatment that can be missed by a single measurement. CGMs provide a successful translation, with the FDA classifying integrated CGM systems as Class II medical devices with special controls when accuracy, safety, and digital connectivity are validated.^163^ However, emerging sweat patches, smart masks, tear sensors, wound dressings, and microneedle biosensors must still demonstrate clinical equivalence and practical value through lower sampling burden, improved monitoring frequency, earlier intervention, and personalized treatment.

### 7.2 Analytical validation under wearable operating conditions

Analytical validation is still inadequate, with many studies citing high sensitivity or low detection limits in buffer, while translation depends on testing conditions under physiological and environmental stress. A new design to translation approach for microneedle electrochemical sensors suggests that rather than direct translation, the sensors should undergo step-wise *in vitro* analytical testing, *ex vivo* skin testing, and *in vivo* validation before clinical use.^136^ This is crucial since after the skin is penetrated, microneedle biosensors need to be electrochemical, reliable, intact, stable, and reproducible.

Comprehensive validation should include sensitivity, selectivity, linear range, LOD, LOQ, real-biofluid recovery, response time, reversibility, reproducibility, storage stability, and operational stability. Testing should match the target biofluid. Sweat sensors should be tested for sweat-rate variations, pH changes, salt concentration, skin contamination, evaporation and temperature, and contact lenses, wound-exudate sensors, and breath-condensate masks demand realistic physiological testing. The stability is essential due to the possibility of degradation, leaching, oxidation, or the loss of selectivity by the enzymes, aptamers, antibodies, MIPs, redox mediators, MXenes, and black phosphorus. Thus, sensors designed for 8 h, 24 h, 7 days, or longer monitoring will require to demonstrate their stability in the presence of mechanical and biochemical stresses.^72,164^

### 7.3 Clinical validation and patient-level reliability

Clinical validation requires direct comparison of wearable biosensor outputs with reference methods in users or patients, but wearable biofluids are not always equivalent to blood. While ISF is clinically relevant for many small molecules, there may be physiological lag, and sweat often has poor sweat–blood correlation and is better suited for hydration, exercise, stress, local physiology, and exposure monitoring unless disease-specific validation is demonstrated.^165^ Tear sensors need to consider reflex tearing and ocular variability, while wound-exudate sensors need to consider spatial heterogeneity.

An electrochemical aptamer patch was developed to monitor vancomycin in Dermal ISF every 5 minutes for 24 h, which would be useful for therapeutic drug monitoring when blood sampling cannot capture pharmacokinetic fluctuations.^144^ Long-term reliability was impaired by sensor degradation, however, and receptor stability and drift control were emphasized. Similarly, a first-in-human microneedle electrochemical biosensor monitored lactate continuously in critically ill and shock-state patients using lactate oxidase and amperometric sensing.^138^ As lactate is a marker of sepsis, hypoxia, shock, and metabolic stress, its clinical use requires increased validation of blood–ISF correlation, physiological lag, performance under edema or poor perfusion, and reliability across ICU populations.

### 7.4 Regulatory readiness, scalable fabrication, and post-deployment safety

Regulatory approval depends strongly on intended use. Wellness devices, like sweat patches for tracking hydration, have less stringent requirements, while devices for drug dosage, insulin control, sepsis detection, or cancer monitoring have more stringent requirements because failing to get a correct reading could impact treatment. This risk-based approach is evident in the FDA classification of integrated CGM systems, where incorrect glucose reading may result in medication administration, thus necessitating additional safety features.^163^

Reproducible manufacturing is key to commercialization. Many prototypes are based on hand-fabricated electrodes, manually assembled microfluidics, laser-induced graphene, SERS substrates, MXenes, hydrogels, printed conductive inks, microneedles, and custom electronics. For scale-up, controlled manufacturing and quality testing, packaging, shelf-life studies, compatibility with the sterilizer, lot-to-lot consistency, and cost control must be studied. Microneedles additionally require fracture-force testing, penetration reproducibility, biocompatibility, sterilization, and safe disposal, while contact lenses and wound dressings require ocular safety, comfort, prolonged tissue contact, and real-use performance.^166–168^

The FreeStyle Libre 3 and Libre 3 Plus recall demonstrates the need for post-market surveillance. In 2026, the FDA identified a Class I recall after certain sensors produced incorrectly low glucose readings, which could lead to delayed insulin dosing or inappropriate carbohydrate intake. The FDA reported 860 serious injuries and seven deaths from even successful commercial wearable biosensors after deployment.^169^

### 7.5 User-centred wearability and data security

User adoption is essential because wearable biosensors must be comfortable, easy to apply, safe to remove, and compatible with daily activities.^170^ Platform-specific usability is also of significant importance. Sweat patches need breathable adhesives and a stable microfluidic contact, microneedle patches must minimize pain and irritation, smart contact lenses need oxygen permeability, optical clarity, comfort, and ocular safety, while wound dressings and textile biosensors must endure long-term wear, replacement, bending, friction, and washing.^171^ If users remove devices early or ignore alerts, strong analytical performance loses clinical value.

Data security is a crucial issue since these devices constantly gather personal and sensitive biochemical data such as glucose levels, fertility hormones, drug levels, alcohol consumption, stress indicators, inflammation markers, wound status, and disease-risk signatures. FDA guidance on cybersecurity focuses on the importance of risk control through the design of the device, the labelling, and the documentation in the premarket submission.^172^ In addition to secure Bluetooth/NFC communication, wearable biosensors must include encrypted cloud transfer, authenticated access, protected firmware updates, and transparent data-ownership policies, especially for pregnancy, reproductive health, drug exposure, alcohol use, stress, and chronic disease biomarkers.

### 7.6 Platform-specific design limitations

Wearable platforms have different design limitations. Sweat biosensors are affected by variable secretion rate, evaporation, skin contamination, and difficult physiological interpretation. A study pointed out that sweat sensing is still hindered by sweat sample collection, sweat generation control, and precise determination of analyte concentration.^165^ Related sampling issues exist for breath-condensate smart masks. The EBCare smart mask achieved real-time breath condensate analysis, but consistent deployment depends on condensation efficiency, humidity control, breathing-pattern regulation and analyte dilution management.^67^

Micro-needle ISF biosensors facilitate systemic biomarker access but face variability in insertion, tissue response, biofouling and physiological lags. A self-calibrating multiplexed microneedle electrode array partially solved these problems by providing single-microneedle resolution and self-calibration logic for glucose, cholesterol, uric acid, lactate, ROS, ions, and pH measurement.^155^ But standardization of insertion depth, biocompatibility and long-term reliability need to be addressed for clinical use.

Fluid heterogeneity, variable exudate volume, bacterial biofilms, inflammatory debris, blood contamination, and dressing pressure are challenges for wound-exudate biosensors. The iCares smart bandage combined pump-free microfluidics with a flexible nanoengineered sensor array to monitor NO, H_2_O_2_, O_2_, pH, and temperature. However, the signal may be strongly influenced by wound stage, infection status, dressing interval and patient mobility, and patient-specific validation is necessary.^143^

### 7.7 Standardized testing and benchmarking

A major limitation is the absence of universal benchmarking standards. Emerging wearable biosensors do not have consistent validation frameworks whereas blood glucose meters and CGMs are evaluated using ISO accuracy criteria, MARD, error grid and clinical accuracy. Recent discussions indicate that MARD alone is not enough and should be interpreted with bias, precision, safety and real-world performance,^173^ as none of these metrics can entirely capture skin adhesion, motion artefacts, biofouling, or population variability.

Standardized reporting should include biofluid source, sampling rate, wearable location, receptor chemistry, transducer type, calibration strategy, LOD, linear range, sensitivity, selectivity, response time, reversibility, drift rate, real-biofluid recovery, temperature effects, mechanical stability, biofouling resistance, shelf life, and wear time. Platform-specific metrics are critical for microneedles, fracture force, insertion depth, pain score, irritation and sterilization data are needed, while for sweat patches and wound bandages, sweat rate, evaporation, contamination, exudate volume, infection status, dressing interval and wound type are important. The eMPatch is a step toward multiplexed AI-assisted benchmarking, as it tracks ISF glucose, uric acid, cholesterol, sodium, potassium, and pH levels, with the machine-learning model yielding 0.996 classification accuracy for metabolic disorder detection and *R*^2^ of 0.977 for severity evaluation.^154^ Broader external validation across varied populations is needed before these claims can be generalized.

### 7.8 Limitations of AI in smart wearable biosensors

Although AI has the potential to enhance noise correction, drift compensation, pattern recognition, multimodal data fusion, and predictive alerts, its value is tied to data quality, sensor reliability, and clinical validation.^174^ Small and homogeneous training sets can result in poor performance when applied to variation in skin tone, age, sex, disease status, medication use, sweat rate, comorbidities, and environmental condition.^175^ If there is no adequate linkage to a clinically verified reference assay, AI could potentially learn device artefacts instead of physiology, resulting in inaccurate biomarker predictions.

The use of AI in wearable biosensors also raises privacy and regulatory issues. FDA guidance for medical devices with embedded AI suggests presetting plans for future model changes, validation, and impact assessments because medical devices may need to be retrained after deployment due to sensor drift, software updates, new patient populations, or larger datasets.^176^ Importantly, AI cannot compensate for unstable recognition chemistry, weak biointerfaces, irrelevant biofluids, or unreliable transduction. Thus, wearable biosensors supported by AI should be validated as integrated systems, sensor chemistry, hardware, algorithms, cybersecurity and clinical workflow before implementation.

## 8 Conclusion and future perspectives

Smart wearable biosensors are making rapid progress from flexible sensing prototypes to fully integrated biochemical monitoring platforms that can continuously monitor sweat, interstitial fluid, tears, wound exudate, breath condensate, and other readily accessible biofluids. In addition to their ability to be miniaturized, they are useful in measuring dynamic changes in biomarker levels that are difficult to measure using traditional laboratory or point-of-care testing, such as metabolic and therapeutic drug monitoring, wound management, hormone and stress profiling, infectious disease detection, cancer surveillance, and personalized healthcare. However, development should come next, beyond proof-of-concept demonstrations and focus on clinical reliability, reproducibility, long-term stability, and clinical use.

Next-generation systems will begin to emphasize self-powered operation, combining biofuel cells, thermoelectric and triboelectric nanogenerators, flexible supercapacitors, and low-power electronics to allow for continuous monitoring without impacting comfort and patient adherence. Meanwhile, self-calibrating biosensors will be necessary to compensate for drift due to biofouling, enzyme degradation, receptor instability, mediator leaching, and temperature or pH changes, which will demand the use of internal reference standards, ratiometric sensing, controlled microfluidics, and algorithm-driven calibration.

The long-term translation will rely on the development of antifouling and biocompatible biointerfaces, in which hydrogels, zwitterionic coatings, porous membranes, nanocomposites, aptamers, antibodies, nanozymes, molecularly imprinted polymers and recognition systems based on CRISPR must be designed as integrated functional systems, not simply as modifications. A new promising field is multiplexed biosensing, in which clinically relevant panels of disease-relevant biomarkers will offer more useful disease assessment than single analyte monitoring. Simultaneously, AI-driven biosensing will revolutionise continuous chemical data by correcting signals, compensating for drift, identifying anomalies, integrating multimodal data, and providing predictive clinical alerts with proper protection of privacy and regulatory approval.

The future of smart wearable biosensors is reliant on the knowledge integration and convergence of chemistry, materials science, bioengineering, microfluidics, electronics, clinical medicine, and AI. Therefore, future wearable biosensors should be assessed not only for their low detection limits or analytical sensitivity but also for their stability, reproducibility, manufacturability, clinical validation, security, comfort and ability to enhance real clinical decisions. The move from a prototyping lab to a clinically proven and trusted, standardized system will shape the future of diagnosis, therapeutic monitoring, and personalized medicine.

## Conflicts of interest

There are no conflicts to declare.

## Data Availability

This article is a review and does not report new experimental or computational data. All data discussed in this study are derived from previously published literature, which has been appropriately cited within the manuscript. No new datasets were generated or analyzed during the current study.
