# Correction: Smart wearable biosensors: a transformative synergy between diagnosis and treatment of disease

**DOI:** 10.1039/d6ra90094a

**Published:** 2026-08-12

**Authors:** Yachana Mishra, Radheshyam Jena, Aman Shukla, Darshan R. Telange, Alaa A. A. Aljabali, Vijay Mishra

**Affiliations:** a School of Bioengineering and Biosciences, Lovely Professional University Phagwara (Punjab)-144411 India; b School of Pharmaceutical Sciences, Lovely Professional University Phagwara (Punjab) 144411 India vijaymishra2@gmail.com; c Datta Meghe College of Pharmacy, Datta Meghe Institute of Higher Education and Research (DMIHER) (DU) Sawangi Meghe Wardha (Maharashtra) 442001 India; d Department of Pharmaceutics & Pharmaceutical Technology, Yarmouk University Irbid Jordan alaaj@yu.edu.jo

## Abstract

Correction for ‘Smart wearable biosensors: a transformative synergy between diagnosis and treatment of disease’ by Yachana Mishra *et al.*, *RSC Adv.*, 2026, https://doi.org/10.1039/d6ra02749k.

The authors regret that the name of one of the authors (Yachana Mishra) was shown incorrectly in the original article. The corrected author list is as shown above.

The Royal Society of Chemistry apologises for these errors and any consequent inconvenience to authors and readers.

